# Psychometric validation of the French Multidimensional Chronic Asthenia Scale (MCAS) in a sample of 621 patients with chronic fatigue

**DOI:** 10.1186/s40359-023-01358-1

**Published:** 2023-10-10

**Authors:** Ingrid Banovic, Fabrizio Scrima, Isabelle Fornasieri, Laurent Beaugerie, Jérémy Coquart, Chloé Fourgon, Pierpaolo Iodice, Isabelle Nion-Larmurier, Guillaume Savoye, Anne-Laure Sorin, Claire Tourny, Maria Augustinova

**Affiliations:** 1 CRFDP Lab., University of Rouen Normandy, 76821 Mont-Saint-Aignan Cedex, France; 2 Faculty of Psychology, University of Strasbourg, 67000 Strasbourg, France; 3 Department of Gastroenterology, Sorbonne Université, INSERM, Institut Pierre Louis d’Epidémiologie Et de Santé Publique, AP-HP, Hôpital Saint-Antoine, 75012 Paris, France; 4 CETAPS Lab., University of Rouen Normandy, Mont-Saint-Aignan, France; 5 Univ. Lille, Univ. Artois, Univ. Littoral Côte d’Opale, ULR - URePSSS - Unité de Recherche Pluridisciplinaire Sport Santé Société, 7369 Lille, France; 6 UMR 10173, Université de Rouen Normandie, Centre Hospitalier Universitaire Charles Nicolle, 76000 Rouen, France

**Keywords:** Health-related quality of life, Chronic asthenia, Fatigue-specific scale, Assessment tool validation

## Abstract

**Background:**

Psychometric validation of the Multidimensional Chronic Asthenia Scale (MCAS) was conducted in order to provide an effective tool for assessing the health-related quality of life of French-speaking patients with chronic asthenia (CA).

**Methods:**

Items resulting from the initial formulation of the self-reported MCAS (along with other materials) were completed by French-speaking volunteers with inactive or active inflammatory bowel disease (IBD-I vs. IBD-A) or chronic fatigue syndrome (CFS). Responses from 621 participants (180 patients with IBD-A, 172 with IBD-I, 269 with CFS) collected in a single online survey were divided into three subsamples to test the construct validity of the MCAS (Step 1, *N* = 240), to confirm its factorial structure (Step 2, *N* = 204) and to explore its convergent-discriminant validity with the Fatigue Symptoms Inventory (FSI) and revised Piper Fatigue Scale (r-PFS, Step 3, *N* = 177).

**Results:**

Steps 1 and 2 showed that, as expected, MCAS has four dimensions: feeling of constraint (FoC), physical (PC), life (LC) and interpersonal consequences (IC), which are also related to the duration of CA (i.e., the longer it lasts, the more the dimensions are impacted). The results further showed that the MCAS is sensitive enough to capture between-group differences, with the CFS group being the most impaired, followed by IBD-A and IBD-I. While convergent-discriminant validity between the 4 factors of MCAS and FSI and r-PFS, respectively, was satisfactory overall, Step 3 also pointed to some limitations that call for future research (e.g., shared variances between the PC and IC dimensions of MCAS and behavioral dimension of r-PFS).

**Conclusion:**

Despite these limitations, the MCAS clearly constitutes a promising tool for measuring quantitative differences (i.e., severity/intensity) in CA associated with various diseases, but also, and importantly, the clinically important differences in domains of its expression (i.e., qualitative differences).

## Background

﻿Although health-related quality of life (HRQoL) in people experiencing fatigue is clearly impaired [1, 2], its assessment is associated with numerous challenges. Indeed, although a large number of fatigue scales exist [3–7], their ability to fully capture this multifaceted and clinically complex construct remains a matter of debate [2].

To illustrate, in healthy individuals in which a lack of vigor or energy and/or decreased motivation to continue a task, associated with feelings of weariness and tiredness [3, 6, 7], was first described [2], this subjective experience usually follows from a period of sustained physical or mental effort and is relieved by rest. ﻿In contrast, fatigue lasting longer than six months (i.e., *chronic fatigue* or *chronic asthenia*, CA) is neither typically related to overexertion nor is it relieved by rest [3, 6, 7]. Although clinically important, CA constitutes a quite unspecific characteristic of more or less transient diseases such as cancer or of chronic diseases such as inflammatory bowel disease (IBD), heart failure or systemic lupus erythematosus, to name just a few [2, 4]. It is also one of the core symptoms of myalgic encephalomyelitis or systemic exercise intolerance disease, also referred to as chronic fatigue syndrome (ME/SEID/CFS) [8–10]. Albeit the etiology of CA associated with this debilitating disease is still poorly understood, it can be severe enough to prevent patients from carrying out their daily activities and/or confine them to bed [8–10], as is the case when CA is associated with malignant diseases. In sum, while both healthy individuals and patients refer to their subjective experience as *fatigue*, interview-based studies suggest that CA is clearly clinically different from fatigue as experienced by healthy individuals [11, 12].

Therefore, while frequency and/or severity/intensity of fatigue (i.e., quantitative differences) within and between these two populations might be efficiently captured by various assessment tools, including those evaluating perceived general health (e.g., [13]), mood (e.g., [14]), or HRQoL (e.g., [15]), these wider-purpose assessment tools may not always be sufficiently sensitive to capture the variability in experiences of fatigue (e.g., physical, affective and cognitive expressions of fatigue) [2, 11, 12] that are referred to here as qualitative differences in fatigue. To address this issue of clinical importance, many fatigue-specific scales have been developed [2], often (but not always) adopting a multidimensional approach, with additional multidimensional scales assessing between two and five dimensions that may comprise the physical, psychological, cognitive, emotional and/or motivational dimensions of fatigue [16].

To make matters even more difficult, measuring disease-related fatigue brings its own additional challenges. While fatigue related to different malignant diseases is alleviated by remission, not only do many ﻿patients continue to experience fatigue but they also report that the fatigue with which they now live is different from the fatigue they experienced before ﻿becoming sick [11, 12]. In IBD, for instance, CA – the origins of which are partly known [17] – persists in nearly half of the patients with inactive IBD (IBD-I; [18, 19]). Since CA is a core and permanent symptom of PE/SEID/CFS, such patients never return to their pre-disease fatigue level and type [20]. Indeed, the severity of fatigue varies from mild (50% reduction of premorbid activity level) to very severe (completely dependent and bedridden). This is due, in particular, to post-exertional malaise (PEM [21, 22]), which patients describe as affecting every part of the body, as being difficult to predict or manage, and as having an unpredictable recovery period requiring complete bedrest for days, weeks or even months [21, 22]. In sum, while there is much variability in IBD-related fatigue (i.e., both within IBD-I and in IBD-I compared to active IBD; IBD-A), much less variability is observed in PE/SEID/CFS. Perhaps as a result of such differences, additional (multidimensional) fatigue-specific scales were designed to evaluate fatigue associated with a given state of a particular disease (e.g., cancer [23], IBD [24] or ME/SEID/CFS [25]).

As a result, Hjollund and colleagues [2] identified 252 different methods for assessing fatigue, of which 150 were used only once. They consequently called for a fatigue-specific assessment tool that sensitively characterizes CA experienced across various diseases. It has been argued [26] that the Fatigue Symptom Inventory (FSI, [27, 28]) is such a tool, as it has been used outside of the field of cancer-related fatigue for which it was first developed. Nevertheless, both IBD (i.e., patients currently considered to experience fatigue similar to that experienced in cancer [29]) and PE/SEID/CFS patients have reported experiences of CA and its consequences that go beyond those captured by the unidimensional FSI [11], especially when IBD is active (i.e., IBD-A).

The present study therefore attempted to validate an assessment tool that possibly captures both quantitative and qualitative differences in disease-related CA more sensitively than the FSI and other fatigue-specific assessment tools available in French. To this end, responses to the *Echelle Multidimensionnelle d’Asthénie Chronique* (Multidimensional Chronic Asthenia Scale, MCAS) – as originally formulated [11] – were collected from IBD (both IBD-A and IBD-I) and PE/SEID/CFS patients in a single online survey. The data from each participant were used in only one step of the validation process. The aim of Step 1 was to assess the construct validity of the MCAS, while Step 2 was designed to verify its factorial structure. Finally, Step 3 had the task of assessing the convergent validity of the MCAS using the FSI [27, 28] and Piper Fatigue Scale (r-PFS) [23], since this latter scale (in the same way as the FSI) was first developed for cancer-related fatigue, but it is now in widespread use. However, its structure (unlike that of the FSI) is clearly multidimensional (in the same way as the target structure for the MCAS), enabling it to measure the behavioral, affective, sensory and cognitive/mood-related expressions of fatigue [11].

## Method

### Recruitment of participants and Ethics

The protocol of the study was approved by the *Comité de Protection des Personnes* (French national ethics committee; CPP2021-02-021b). A total of 818 individuals volunteered to take part in the study in response to an advertisement issued by two patient associations – Association François Aupetit (AFA) and Association Française du Syndrome de Fatigue Chronique (ASFC). Before agreeing to participate in this study (which took the form of a single online survey administered between June 2021and January 2022), the participants first read a Participant Information Sheet containing all the information about the present study. They could not access the questionnaire, until they had given their (fully informed) consent by ticking the appropriate response box that the online Participant Information Sheet.

### Inclusion criteria and resulting sample

The inclusion criteria were (1) age 18–60 years; (2) fatigue lasting more than 6 months; and (3) diagnosis of IBD or ME/SEID/CFS. After excluding incomplete questionnaires (*N* = 183) and those of participants who did not meet these criteria (*N* = 14), the data of 621 participants were finally retained (486 identified themselves as female and 135 as male). It should be noted that this gender disbalance is due to both higher prevalence of CA in women [30] and high response rates that women display in online surveys [31] (i.e., type of survey used in the present study). This overall sample was divided into 3 distinct subsamples used for separate analyses conducted in Step 1 (*N* = 240), Step 2 (*N* = 204) and Step 3 (*N* = 177) of the MCAS psychometric validation process. In other words, the data from each participant (subsampled in a pseudo-random manner) were used in only one step of this process.

### Materials included in the study

#### Demographic and medical condition data

The participants first responded to a short questionnaire asking about their age, gender, education level, marital status, professional activity, date of the IBD or ME/SEID/CFS diagnosis, duration of the fatigue state and current fatigue level. The IBD patients were also asked to report the type of IBD. In Crohn’s disease patients, disease activity was subsequently assessed using the Harvey–Bradshaw Index [32]. For patients with ulcerative colitis, this information was collected using the Simple Clinical Colitis Activity Index [33]. A disease activity level of 4 or below was classified as IBD-I, and a level of 5 and above was classed as IBD-A (see Table 1).

**Table 1:** Sociodemographic characteristics of sub-samples used in Steps 1–3 of the validation process

	Step 1	Step 2	Step 3
N	240	204	177
Women	195 (81.3%)	155 (76%)	136 (76.8%)
Men	45 (18.8%)	49 (24%)	41 (23.2%)
IBD-A	60 (25%)	62 (30.4%)	58 (33.3%)
IBD-I	58 (24.2%)	59 (28.9%)	54 (30.5%)
SFC	122 (50.8%)	83 (40.7%)	64 (36.2%)
	Mean (SD)	Mean (SD)	Mean (SD)
Age	39.52 (10.41)	39.82 (10.59)	40.50 (10.86)
Fatigue duration in months	107.43 (97.58)	90.90 (84.89)	74.30 (76.27)
FSI	85.65 (18.14)	79.65 (15.94)	75.34 (18.05)
FSI—Perturbation	45.45 (11.64)	42.22 (10.49)	39.58 (12.18)
HAD-A	10.20 (4.27)	9.75 (3.97)	-
HAD-D	9.65 (3.83)	9.19 (3.47)	-
r-PFS Total score	7.67 (1.53)	7.49 (1.51)	6.81 (1.45)
r-PFS Affective	8.27 (1.49)	7.90 (1.69)	7.67 (1.83)
r-PFS Sensory	7.65 (1.45)	7.48 (1.59)	6.71 (1.64)
r-PFS Cognitive	6.27, (1.67)	7.10 (1.61)	5.26 (1.56)
r-PFS Behavioral	9.34 (1.89)	9.11 (1.97)	7.24 (1.56)
TAS-20 Total score	56.83 (12.69)	57.55 (10.91)	-
TAS-20 Description	14.53 (4.68)	14.73 (4.25)	-
TAS-20 Identification	22.77 (6.24)	23.26 (5.28)	-
TAS-20 Operational thinking	19.53 (4.68)	19.56 (3.96)	-

#### Fatigue assessment data

The FSI was administered first. Responses to fourteen items of its French version [34] were scored as in [27, 28], and all responses were summed to yield a total score, while responses to seven interference items were summed to yield a global interference score, with higher scores indicating a greater impact of CA (see Table 1 for averaged sums of both scores).

Fatigue was then evaluated using the MCAS with a reflective approach. Each of the 20 item – resulting from the initial formulation of the self-reported MCAS [11] (see Table 2) – was assessed on an 11-point rating scale ranging from 0 (never) to 10 (always). The participants were instructed to respond as honestly and as accurately as possible. Higher mean scores indicate a greater impact of CA on each of the four dimensions in experience of fatigue that commonly emerged during the initial MCAS formulation [11] (i.e., MCAS-Feeling of Constraint (MCAS-FoC); MCAS-Interpersonal Consequences of chronic asthenia (MCAS-IC); MCAS-Daily Life Consequences of chronic asthenia (MCAS-LC) and MCAS-Physical Consequences of chronic asthenia (MCAS-PC)). Means for each item and/or averaged means for each dimension (see Table 5) were used for analyses reported here below.

**Table 2:** Exploratory factor analysis conducted in Step 1 of the validation process

	Feeling of constraint	Physical consequences	Life consequences		Interpersonal consequences
*La fatigue m'a contraint (e) à : réduire/réaménager mes activités professionnelles* (Fatigue has constrained me to: reduce/reorganize my work activities)	.825				
*La fatigue m'a contraint (e) à : réorganiser mon rythme de vie* (Fatigue has constrained me to: reorganize my life routine)	.804				
*La fatigue m'a contraint (e) à : réduire mes activités quotidiennes et mes loisirs (et pas seulement à les aménager)* (Fatigue *has constrained* me to: to reduce my daily activities and leisure time (not just to adjust them))	.795				
*La diminution de mes activités est uniquement liée à la fatigue et pas uniquement à du désintérêt* The reduction in my activities is only related to fatigue and not to lack of interest	.606				
*La fatigue se manifeste essentiellement de façon corporelle (muscles, articulations, sensations corporelles)* (Fatigue is mainly experienced in the body (muscles, joints, body sensations))		.768			
*Cette fatigue est la conséquence de douleurs musculaires et/ou articulaires, de douleurs physiques* (This fatigue is the consequence of muscular and/or articular pains, physical pains)		.565			
*La fatigue ressemble à un « état grippal » (fièvre, douleurs, courbatures, brouillard intellectuel)* (Fatigue resembles a "flu-like condition" (fever, aches, pains, mental fogginess))		.388			
*La fatigue m’empêche de réaliser des choses de la vie courante (comme par exemple préparer un repas en semaine)* (Fatigue impedes me from doing everyday things (such as preparing a meal during the week))			.855		
*La fatigue entraîne une difficulté à commencer et accomplir des activités quotidiennes* (Fatigue makes it difficult to start and complete daily activities)			.646		
En ce moment, lorsque je fais quelque chose de simple (un repas en semaine), les conséquences sur la fatigue sont… (At this time, when I do something simple (a weekday meal), the impact on fatigue is...)			.609		
*La fatigue m’empêche de me concentrer, de faire des activités impliquant de l’attention.* (Fatigue impairs my ability to concentrate, to do activities that require attention).			.489		
*La fatigue a des conséquences sur les moments passés avec d’autres personnes (amis, famille, etc.)* (Fatigue has consequences on the time spent with other people (friends, family, etc.))					.972
*La fatigue est une souffrance pour moi.* (Fatigue is a suffering for me.)					.690
*La fatigue interfère dans mes relations avec les personnes.* (Fatigue interferes with my interactions with people.)					.608
Explained variance	18.55%	7.88%	14.20%		14.45 %
Alpha	.85	.58	.78		.84

*N* 240, Extraction Method, Principal Axis Factoring, Rotation Method, Oblimin with Kaiser Normalization, Rotation converged in 7 iterations

Fatigue was then finally evaluated using the r-PFS [23]. Responses to twenty-two items from the French version [35] were scored as in [35], and all responses were averaged to yield a total score. Similarly, responses were averaged to yield a total score for each of the four dimensions (i.e., behavioral, affective, sensory and cognitive/mood-related consequences of fatigue). Again, higher mean scores indicate a greater impact of CA in general or on each dimension (see Table 1 for these averaged means).

#### Broader psychological evaluation data

Because both depression and altered emotional processing play a role in CA [36], the French version [37] of the Hospital Anxiety and Depression Scale (HADS; [38]) was administered to assess both anxiety (HAD-A) and depression (HAD-D), with higher scores on these two subscales indicating higher levels of anxiety and depression, respectively (with an anxiety score above 12 points corresponding to pathological anxiety level and a depression score above 8 points corresponding to clinal depression, see Table 1 for these averaged sums).

Emotional processing was assessed using the French version [39] of the Toronto Alexithymia Scale (TAS-20, [40]). All responses were summed to yield a total score (TAS-20 Total score), with higher scores indicating a higher probability of alexithymia (i.e., scores between 52 and 60 indicating possible alexithymia, and scores above 61 indicating alexithymia). As in [39], responses to five items were summed to assess difficulty identifying feelings (TAS-20 Identification), those to seven items were summed to assess difficulty describing emotions (TAS-20 Description) and those to eight items were summed to assess the ability of individuals to focus their attention externally (TAS-20 Operational thinking, see Table 1 for all averaged sums).

In sum, the questionnaires and scales listed above were administered in a fixed order, with the demographic and medical condition questionnaire being followed by the FSI [34], HADS [37], MCAS [11], TAS-20 [39] and r-PFS [35].

## Results

### Step 1: construct validity of MCAS

To assess the construct validity, responses to MCAS with 20 items of 240 pseudorandomly sampled patients (81.2% female, 18.8% male; M_age_ = 39.52 ± 10.41) – 25% of whom were affected by IBD-A, 24.2% by IBD-I and the remaining 50.8% by ME/SEID/CFS (see Table 1) – were analyzed by means of an exploratory factor analysis (EFA) using the Principal Axis Factoring method. The sample size adequacy was determined by means of the KMO index and the mean communality, resulting in 0.85 and 0.56 respectively. The matrix underwent a Direct-Oblimin rotation with Kaiser Normalization. The number of factors to be extracted was determined based on an eigenvalue greater than 1 and the Bartlett’s scree test. All factorial weights below.30 were eliminated. Specifically, the criteria for keeping the items were as follows: saturation on a single factor, saturation greater than 0.30, and semantic coherence between item and factor.

As shown in Table 2, this method permitted the extraction of 4 factors explaining a total of 55.08% of the variance. The first factor is characterized by 4 items describing the feeling of constraint. The second factor consists of 3 items describing the physical consequences. The third factor (4 items) describes the physical consequences, and the fourth factor (three items) describes the interpersonal consequences. Item saturations fall within a range of 0.388 to 0.972.

An additional analysis of variance and post hoc Dunnett’s T3 test were used to compare the scores obtained on each factor among the 3 groups (IBD-A, IBD-I, ME/SEID/CFS). These analyses indicated a significant difference in the scores obtained in three out of four factors in IBD-A, IBD-I and ME/SEID/CFS patients. As shown in Tables 3 and 4, ME/SEID/CFS patients obtained higher mean MCAS-FoC scores than IBD-A (*p* < 0.001) and IBD-I patients (*p* < 0.001). No significant difference was present for MCAS-PC. Regarding MCAS-LC, ME/SEID/CFS patients obtained higher mean scores than IBD-A (*p* = 0.03) and IBD-I patients (*p* < 0.001). To conclude, the same trend was found in MCAS-IC, with ME/SEID/CFS patients scoring higher than IBD-A (*p* = 0.002) and IBD-I (*p* = 0.002) patients.

**Table 3:** Results of ANOVA with post hoc Dunnett T3 test conducted in Step 1 of the validation process

	IBD-A (*N* = 60)		IBD-I (*N* = 58)		ME/SEID/CFS (*N* = 122)					
	Mean	(SD)	Mean	(SD)	Mean	(SD)	F	*p*	Eta^2^	Interpretation
MCAS-FoC	7.14	2.02	6.97	2.67	9.25	1.18	41.88	< .001	.26	Large
MCAS-PC	6.30	2.25	5.87	2.57	5.93	2.23	.63	.534	.00	Small
MCAS-LC	6.59	2.16	5.82	2.06	7.34	1.82	12.36	< .001	.09	Medium
MCAS-IC	7.76	1.65	7.45	2.29	8.63	1.48	10.61	< .001	.08	Medium

**Table 4:** Results of ANOVA with post hoc Dunnett T3 test conducted in Step 1 of the validation process

		MCAS-FoC	MCAS-PC	MCAS-LC	MCAS-IC
Comparisons		*p*	*p*	*p*	*p*
IBD-A	IBD-I	.973	.713	.235	.800
IBD-A	SFC	< .001	.662	.028	.002
IBD-I	SFC	< .001	.998	< .001	.002

*FOC* Feeling of constraint, *PC* Physical consequences, *LC* Life consequences, *IC* Interpersonal consequences

### Step 2: factorial structure of MCAS

The aim of Step 2 was to confirm the factorial structure of the MCAS using confirmatory factor analysis. To this end, responses to the MCAS of 204 pseudorandomly sampled patients (76% female, 24% male; M_age_ = 39.82 ± 10.59) – 30.4% of whom were affected by IBD-A, 28.9% by IBD-I and the remaining 40.7% by ME/SEID/CFS (see Table 1) – were used to verify the scale’s factorial structure. A post hoc power analysis, based on MacCallum et al.’s recommendations [41], was performed to check the appropriateness of the sample size. The analysis showed that with a sample size of 204 subjects we obtain a power of 0.81. A confirmatory factor analysis of the factorial structure revealed by the first study was first performed on these responses using structural equation models (SEM). The Maximum Likelihood extraction method was used. The following fit indices were used to evaluate the goodness of fit of the models: Carmines-McIver index $\chi$ ^2^/df), Comparative Fit Index (CFI), Tucker – Lewis Non-Normed Fit Index (NNFI) and the Root Mean Square Error of Approximation (RMSEA). A Χ^2^/df ratio of 3 to 1 indicates a good fit of the collected data [42]. Following [43], threshold values greater than or equal to 0.90 for CFI and NNFI and values lower than or equal to 0.06 for RMSEA were applied for the remaining indices. The comparison between models was tested using the $\Delta \chi$ ^2^ test.

Three models were compared to confirm the factorial structure. In the first model, all items saturated on a single latent factor. In the second model, four latent factors were created based on the item saturation levels indicated by the EFA. The third model exactly mirrors the factorial solution of the exploratory analysis, i.e., four correlated latent factors. As shown in Table 5, the correlated 4-factor model is the only model that shows satisfactory fit indices.

**Table 5:** Results of confirmatory factor analysis (Comparison of 3 models) conducted in Step 2 of the validation process

Model	$\chi$ ^2^	df	*p*	$\chi$ ^2^/df	CFI	NNFI	RMSEA (LO)(HI)
One-factor	434	76	< .001	5.72	.71	.65	.15 (.14)(.17)
Four-factor	310	76	< .001	4.08	.81	.77	.12 (.11)(.14)
Correlated four-factor	127	70	< .001	1.81	.95	.94	.06 (.05)(.08)

Correlation coefficients between MCAS dimensions, gender, duration of fatigue in months, age, two fatigue scales (FSI and r-PFS), HAD and TAS-20 were then calculated. As indicated in Table 6, the MCAS-FoC had significant positive correlations (all *p*_*s*_ < 0.01 unless indicated otherwise) with age, FSI, and HAD-A in all dimensions of r-PFS except the cognitive dimension (*r* = 0.01, *p* = 0.94). MCAS-PC exhibited significant positive correlations with (all *p*_*s*_ < 0.01 unless indicated otherwise) sex, FSI, and the behavioral dimension of r-PFS (*p* < 0.05). The MCAS-LC had significant positive correlations (all *p*_*s*_ < 0.01 unless indicated otherwise) with sex, FSI, HAD-D, all dimensions of r-PFS, and TAS-20 operational thinking (*p* = 0.03). Furthermore, the MCAS-IC exhibited significant positive correlations (all *p*_*s*_ < 0.01) with FSI, HAD-D, and all dimensions of r-PFS. Finally, due to the gender disbalance, the differences between the scores in the 4 dimensions of the MCAS of individuals identifying as males and females respectively were also investigated by means of an analysis of variance. However, the results indicated that no statistically significant differences were found (all *p*_*s*_ < 0.05).

**Table 6:** Correlation matrix computed in Step 2 of the validation process

		1	2	3	4	5	6	7	8	9	10	11	12	13	14	15	16	17	18
1	Sex	1																	
2	Fatigue duration in months	.04	1																
3	Age	-.031	.347^b^	1															
4	FSI	-.154^a^	.081	.019	1														
5	HAD-A	-.138^a^	-.103	-.146^a^	.09	1													
6	HAD-D	-.140^a^	-.054	.081	.386^b^	.346^b^	1												
7	r-PFS Total score	-.316^b^	.033	.113	.574^b^	.103	.485^b^	1											
8	r-PFS Affective	-.117	.004	.064	.527^b^	-.088	.326^b^	.872^b^	1										
9	r-PFS Sensory	-.183^b^	-.023	.015	.484^b^	.143^a^	.456^b^	.791^b^	.627^b^	1									
10	r-PFS Cognitive	-.153^a^	-.069	.051	.359^b^	.399^b^	.531^b^	.659^b^	.461^b^	.609^b^	1								
11	r-PFS Behavioral	-.181^b^	.059	.142^a^	.595^b^	-.001	.446^b^	.804^b^	.769^b^	.635^b^	.412^b^	1							
12	TAS-20 Total score	.034	-.224^b^	-.253^b^	-.043	.398^b^	.223^b^	.081	-.012	.107	.274^b^	.045	1						
13	TAS-20 Identification	-.048	-.235^b^	-.242^b^	-.004	.464^b^	.264^b^	.149^a^	.041	.163^a^	.301^b^	.119	.848^b^	1					
14	TAS-20 Description	.061	-.125	-.241^b^	-.025	.225^b^	.206^b^	.069	-.025	.086	.158^a^	.042	.834^b^	.590^b^	1				
15	TAS-20 Operational thinking	.089	-.168^a^	-.114	-.085	.235^b^	.04	-.048	-.06	-.015	.181^b^	-.077	.714^b^	.368^b^	.423^b^	1			
16	MCAS-FoC	-.102	.111	.259^b^	.311^b^	-.224^b^	.121	.308^b^	.285^b^	.188^b^	.006	.470^b^	-.240^b^	-.175^a^	-.175^a^	-.235^b^	1		
17	MACS-PC	-.144^a^	.119	.011	.260^b^	-.015	.046	.116	.118	.106	.046	.139^a^	-.066	-.055	-.028	-.076	.245^b^	1	
18	MCAS-LC	-.196^b^	.067	.031	.589^b^	-.086	.296^b^	.491^b^	.388^b^	.351^b^	.254^b^	.564^b^	-.089	-.009	-.072	-.152^a^	.556^b^	.299^b^	1
19	MCAS-IC	-.122	.015	-.053	.413^b^	.1	.380^b^	.449^b^	.276^b^	.396^b^	.253^b^	.571^b^	.006	.046	.035	-.079	.423^b^	.094	.570^b^

*N* 204,^a^ Correlation is significant at the .05 level (2-tailed),^b^ Correlation is significant at the. 01 level (2-tailed), *MCAS-FOC* Feeling of constraint, *MCAS-PC* Physical consequences, *MCAS-LC* Life consequences, *MCAS-IC* Interpersonal consequences, *EOT* Externally Oriented Thinking, *HAD-A* Hospital Anxiety Depression Scale-Anxiety, *HAD-D* Hospital Anxiety Depression Scale-Depression

As in Step 1, an additional analysis of variance and post hoc Dunnett’s T3 test were used to test the difference between the scores in the three groups as had already been done in the first study. As indicated in Table 7, the results of these analyses confirmed those of Step 1 for the factors MCAS-FoC and MCAS-PC. ME/SEID/CFS patients obtained higher MCAS-FoC scores than IBD-A (*p* < 0.001) and IBD-I (*p* < 0.001) patients. There was no significant difference for MCAS-PC. Additionally, ME/SEID/CFS patients obtained higher MCAS-IC scores than IBD-I patients (*p* < 0.001), and IBD-A patients also obtained higher scores than IBD-I patients (*p* = 0.037). However, there was no significant difference between IBD-A and ME/SEID/CFS patients. Finally, ME/SEID/CFS patients also obtained higher MCAS-LC scores than IBD-A (*p* = 0.004) and IBD-I patients (*p* < 0.001).

**Table 7:** Results of ANOVA with post hoc Dunnett T3 test conducted in Step 2 of the validation process

	IBD-A (*N* = 62)		IBD-I (*N* = 59)		SFC (*N* = 83)					
	Mean	(SD)	Mean	(SD)	Mean	(SD)	F	p	Eta^2^	Interpretation
MCAS-FoC	7.62	1.97	5.94	2.71	9.32	1.00	53.76	< .001	.35	Large
MCAS-PC	5..47	2.25	5.06	2.03	5.53	2.18	.91	.403	.00	Small
MCAS-LC	6.35	1.54	5.39	1.96	7.35	1.53	26.16	< .001	.21	Large
MCAS-IC	8.10	1.57	7.19	1.86	8.63	1.33	10.98	< .001	.13	Large

### Step 3: convergent validity of MCAS

The aim of Step 3 was to test the convergent and discriminant validity between the MCAS, FSI and r-PSF. To this end, the responses to these scales of 177 pseudo-randomly sampled patients (76,8% female, 23,2% male; M_age_ = 40.50 ± 10.86) – 33.3% of whom were affected by IBD-A, 28.9% by IBD-I and the remaining 36.2% by ME/SEID/CFS (see Table 1) – were analyzed. A post hoc power analysis [41], was performed to check the appropriateness of the sample size. The analysis showed that with a sample size of 177 subjects, we obtain a power of 0.99. Following [44], Structural Equation Models (SEM) were used to compare the shared variance between latent variables with the average variance extracted (AVE) of each variable. As far as the convergent-discriminant validity between the 4 factors of MCAS and FSI is concerned, the results indicate that the correlated 4-factor model is significantly better than the single-factor solution ($\Delta \chi$ ^2^ = 370, $\Delta$_df_ = 10, *p* < 0.001). All items show a factorial weight ranging from 0.30 to 0.97 and were significant (*p* < 0.001). Tables 7 and 8 shows that no shared variance between FSI and the 4 factors of the MCAS was higher than the average variance extracted, indicating adequate discrimination of the constructs.

**Table 8:** Results of ANOVA with post hoc Dunnett T3 test conducted in Step 2 of the validation process

		MCAS-FoC	MCAS-PC	MCAS-LC	MCAS-IC
Comparisons		*p*	*p*	*p*	*p*
IBD-A	IBD-I	.001	.636	.004	.037
IBD-A	SFC	< .001	.998	.001	.165
IBD-I	SFC	< .001	.464	< .001	< .001

Table 9 shows the results of the convergent-discriminant analysis between 4 factors of the MCAS and 4 factors of the r-PFS. Again, the 8-factor correlated model is significantly better than the single-factor solution ($\Delta \chi$ ^2^ = 1030, $\Delta$_df_ = 28, *p* < 0.001). Table 9 shows that all shared variances were lower than the average variance extracted. However, it is worth noting that the analysis recorded high shared variances between r-PFS Behavioral and both MCAS-PC (SV = 0.52) and MCAS-IC (SV = 0.62).

**Table 9:** Results of convergent-discriminant analysis between MCAS and FSI conducted in Step 3 of the validation process

	Average variance extracted	Shared variance with FSI
	MCAS	
MCAS-FoC	.78	.42
MCAS-PC	.53	.17
MCAS-LC	.76	.59
MCAS-IC	.75	.37

To test the predictivity of the 4 dimensions of the MCAS, two ROC analyses were performed. The criterion variable was the total score on the r-PFS scale. The first analysis investigated the predictive validity of positive cases with moderate and severe fatigue scores (cutoff ≥ 4, see Tables 10, 11, 12). The second analysis investigated this issue with severe fatigue scores (cutoff ≥ 7, see Tables 10, 11, 12) [45]. The results of these analyses indicate that all 4 factors have predictive ability for moderate and severe fatigue levels.

**Table 10:** Results of convergent-discriminant analysis between MCAS and r-PFS conducted in Step 3 of the validation process

	Average variance extracted	Shared variance with:			
	MCAS	r-PFS Behavioral	r-PFS Affective	r-PFS Sensory	r-PFS Cognitive
MCAS-FoC	.78	.31	.15	.03	.04
MCAS-PC	.53	.52	.22	.16	.23
MCAS-LC	.76	.06	.04	.03	.06
MCAS-IC	.75	.62	.28	.11	.06

**Table 11:** ROC analysis with r-PFS scores ≥ 4 (moderate level of fatigue) conducted in Step 3 of the validation process

	AUC	S.E	*p*	LLCI	ULCI
MCAS-FoC	.721	.044	< .001	.635	.867
MCAS-PC	.601	.049	.039	.505	.697
MCAS-LC	.809	.036	< .001	.739	.879
MCAS-IC	.823	.039	< .001	.755	.891

Criterion variable: r-PFS total score, Cutoff score ≥ 4 (moderate fatigue level), *Positive cases* 128, *Negative cases* 74, *AUC* Area Under Curve, SE = Standard error, *LLCI* Lower limit confidence interval, *ULCI* Upper limit confidence interval

**Table 12:** ROC analysis with r-PFS scores ≥ 7 (severe level of fatigue) conducted in Step 3 of the validation process

	AUC	S.E	*p*	LLCI	ULCI
MCAS-FoC	.640	.042	= .001	.557	.722
MCAS-PC	.595	.043	.030	.510	.679
MCAS-LC	.759	.036	< .001	.689	.829
MCAS-IC	.749	.037	< .001	.678	.821

Criterion variable: r-PFS total score, Cutoff score ≥ 7 (severe fatigue level), *Positive cases* 82, *Negative cases* 95, *AUC* Area Under Curve, *SE* Standard error, *LLCI* Lower limit confidence interval, *ULCI* Upper limit confidence interval

## Discussion

As CA is unlikely to be satisfactorily described on a simple continuum going from no to severe fatigue [2, 11, 36, 46], the present study undertook a psychometric validation of the Multidimensional Chronic Asthenia Scale to better apprehend patients’ quality of life in disease-related CA.

Step 1 of the MCAS psychometric validation process was specifically designed to test the construct validity. Step 2 was designed to verify the MCAS factorial structure. ﻿The results both confirmed the scale’s construct validity and validated its factorial structure. Indeed, the reported analyses converge toward the idea that MCAS has four factors: feeling of constraint (caused by CA), physical consequences, life consequences and interpersonal consequences (of CA) directly mapping onto four dimensions in experience of fatigue that commonly emerged during the initial MCAS formulation [11].

Importantly and in line with past findings [47], these four dimensions are related to the duration of CA such that the longer it lasts, the more the dimensions are impacted. This latter result suggests that, in spite of its clear chronicity (i.e., at the time of inclusion, all the participants had been experiencing CA for at least 6 months), CA continues to evolve, and MCAS is likely to be sensitive enough to capture the qualitative differences that this evolution entails.

Importantly, Steps 1 and 2 of the validation process also revealed that the four dimensions of the MCAS differed significantly across the clinical groups involved in the study. In line with past findings [11, 48], ME/SEID/CFS patients were the most impaired by CA, followed by IBD-A and IBD-I patients. The fact that MCAS captures different degrees of CA severity that occur as a function of different chronic diseases (ME/SEID/CFS vs. IBD) and/or of the patients’ specific state (IBD-A vs. IBD-I) shows that it is clearly sensitive to the particular context in which CA occurs. Finally, and again in line with past studies showing that anxiety and depression contribute to the persistence of CA [34, 46, 47, 49], the present study also found a strong positive association between the MCAS and the HADS depression scale (HAD-D) [37, 38].

Step 3 of the validation process was designed to examine the convergent-discriminant validity of the MCAS. Although Step 2 found a strong positive association between the MCAS and the two validated fatigue-specific scales (i.e., FSI [27, 28, 34] and r-PFS [23, 35]), Step 3 also revealed a significant difference between the MCAS and FSI and r-PFS. Finally, the results of the two ROC analyses showed that the 4 factors of MCAS have predictive ability for moderate and severe fatigue levels. However, while the convergent-discriminant validity between the 4 factors of MCAS and FSI and r-PFS was satisfactory overall, some limitations were also revealed at Step 3 of the validation process. Indeed, the r-PFS Behavioral dimension – which uses six items to assess the intensity of fatigue and its impact on physical and social activities – seems very similar to the MCAS-PC and MCAS-IC. This is not particularly surprising given that the 3-item MCAS-PC similarly assesses the physical consequences of CA and the 4-item MCAS-IC assesses the interpersonal consequences of CA. While MCAS might therefore appear to be less parsimonious than r-PFS, it should be remembered here that all shared variances were lower than the average variance extracted for MCAS. Another limitation is that the MCAS-PC also yielded insufficient convergent validity (a = 0.60). Although a short scale is often easier for people to complete, adding items for this dimension might therefore appear as necessary, as this would probably enhance the internal consistency of the items.

### Future directions and concluding remarks

Future studies using the MCAS need to address the issue of internal consistency raised above. While, as reported above, the MCAS is likely to be sensitive to qualitative differences brought about by the evolution of CA over time, it seems necessary to test this sensitivity to change further. These further tests should include repeated administration(s) of the MCAS to the same participants in distinct clinical groups. Such research will also likely improve the accuracy of MCAS in identifying the consequences (in nature and degree) that are the most sensitive to variations in the severity of CA [50]. As has previously been shown for chronic pain [51], the chronicity of fatigue is likely to influence the perception of its consequences due to both conditioning and memory mechanisms [47, 52]. Because the disappearance of these learned responses is slow and highly dependent on inhibitory mechanisms [47], future studies also need to examine the extent to which inhibition – as measured by means of a more fine-grained Stroop task [53, 54] – is impaired in individuals with CA (both independently and as a function of CA severity). Using MCAS in studies of this type will make it possible to establish whether the subjectively reported type and level of CA are linked to objective cognitive performances in such a way that MCAS scores are likely to predict individuals’ actual overall cognitive performance as measured by specific components of the Stroop effect [55] that are generated by the Stroop task [53, 54]. In the meantime, despite the various limitations outlined above, MCAS clearly constitutes a promising tool for measuring in clinical practice both quantitative and qualitative differences in CA associated with various diseases [2]. This instrument not only provides a standardized assessment of CA, but also represents a support instrument for a more specific patient-clinician communication, namely that about the clinically important differences in the expression of this complex phenomenon.

## Data Availability

The datasets used and/or analyzed during the current study are available from the corresponding author on reasonable request.

## Abbreviations

AFA: *Association François Aupetit Chronique* (French association of IBD patients)
ASFC: *Association Française du Syndrome de Fatigue Chronique* (French association of ME/SEID/CFS patients)
AVE: Average variance extracted
CA: Chronic asthenia
CFI: Comparative fit index
EFA: Exploratory factor analysis
FSI: Fatigue symptom inventory
HAD-A: Hospital anxiety subscale
HAD-D: Hospital depression subscale
HADS: Hospital anxiety and depression scale
IBD-A: Active inflammatory bowel disease
IBD-I: Inactive inflammatory bowel disease
IBD: Inflammatory bowel Disease
KMO: Kaiser–Meyer–Olkin test for sampling adequacy
MCAS: Multidimensional chronic asthenia scale
MCAS-FoC: MCAS-feeling of constraint
MCAS-IC: MCAS-Interpersonal consequences of chronic asthenia
MCAS-LC: MCAS-Daily life consequences of chronic asthenia
MCAS-PC: MCAS-Physical consequences of chronic asthenia
ME/SEID/CFS: Myalgic encephalomyelitis/systemic exercise intolerance disease/chronic fatigue syndrome
NNFI: Lewis Non-Normed fit index
PEM: Post-Exertional malaise
r-PFS: Revised piper fatigue scale
RMSEA: Root mean square error of approximation
ROC analysis: Receiver operating characteristic analysis
SEM: Structural equation models
TAS-20: Toronto alexithymia scale

## References

[1] Dukes JC, Chakan M, Mills A, Marcaurd M (2021). Approach to Fatigue: Best Practice. Med Clin N.

[2] Hjollund NH, Hviid Andersen J, Bech P (2007). Assessment of fatigue in chronic disease: a bibliographic study of fatigue measurement scales. Health Qual. Life Outcomes.

[3] Aaronson LS, Teel CS, Cassmeyer V, Neuberger GB, Pallikkathayil L, Pierce J, Press AN, Williams PD, Wingate A (1999). Defining and measuring fatigue. Image J Nurs Sch.

[4] Ahlberg K, Ekman T, Gaston-Johansson F, Mock V (2003). Assessment and management of cancer-related fatigue in adults. Lancet.

[5] Dittner AJ, Wessely SC, Brown RG (2004). The assessment of fatigue: a practical guide for clinicians and researchers. J Psychosom Res.

[6] Finsterer J, Mahjoub SZ (2014). Fatigue in healthy and diseased individuals. Am J Hosp Palliat Care.

[7] Jason LA, Evans M, Brown M, Porter N (2010). What Is Fatigue?. Pathological Nonpathological Fatigue PMR.

[8] Lim EJ, Son CG (2020). Review of case definitions for myalgic encephalomyelitis/chronic fatigue syndrome (ME/CFS). J Transl Med.

[9] Deumer US, Varesi A, Floris V, Savioli G, Mantovani E, López-Carrasco P, Rosati GM, Prasad S, Ricevuti G (2021). Myalgic encephalomyelitis/Chronic FATIGUE SYNdrome (ME/CFS): an overview. J Clin Med.

[10] Corbitt M, Eaton-Fitch N, Staines D, Cabanas H, Marshall-Gradisnik S (2019). A systematic review of cytokines in chronic fatigue syndrome/myalgic encephalomyelitis/systemic exertion intolerance disease (CFS/ME/SEID). BMC Neurol.

[11] Fourgon C, Sorin A-L, Fornasieri I, Scrima F, Tourny C, Coquart J, Nion-Larmurier I, Augustinova M, Banovic I. The chronic fatigue experience of patients with CFS/ME or with IBD and general population subjects: A quantitative and qualitative analysis of the discourse before the design of a chronic asthenia scale. Ann Med Psychol. 2022; online first. 10.1016/j.amp.2022.07.005.

[12] Glaus A, Crow R, Hammond S (1996). A qualitative study to explore the concept of fatigue/tiredness in cancer patients and in healthy individuals. Eur J Cancer Care.

[13] Wiklund I (1990). The Nottingham health profile: a measure of health-related quality of life. Scand J Prim Health Care.

[14] Denollet J, De Vries J (2006). Positive and negative affect within the realm of depression, stress and fatigue: the two-factor distress model of the Global Mood Scale (GMS). J Affect Disord.

[15] Harel D, Thomb B, Hudson M, Baron M, Steele R, and on behalf of Canadian Scleroderma Research Group. Measuring fatigue in SSc: a comparison of the Short Form-36 Vitality subscale and Functional Assessment of Chronic Illness Therapy–Fatigue scale. Rheumatology. 2012; 51(12): 2177 -2185. 10.1093/rheumatology/kes206 10.1093/rheumatology/kes206 22923749

[16] Hewlett S, Dures E, Almeida C (2011). Measures of Fatigue: Bristol Rheumatoid Arthritis Fatigue Multi-Dimensional Questionnaire (BRAF MDQ), Bristol Rheumatoid Arthritis Fatigue Numerical Rating Scales (BRAF NRS) for Severity, Effect, and Coping, Chalder Fatigue Questionnaire (CFQ), Checklist Individual Strength (CIS20R and CIS8R), Fatigue Severity Scale (FSS), Functional Assessment Chronic Illness Therapy (Fatigue) (FACIT-F), Multi-Dimensional Assessment of Fatigue (MAF), Multi-Dimensional Fatigue Inventory (MFI), Pediatric Quality Of Life (PedsQL) Multi-Dimensional Fatigue Scale, Profile of Fatigue (ProF), Short Form 36 Vitality Subscale (SF-36 VT), and Visual Analog Scales (VAS). Arthritis Care Res.

[17] McGing JJ, Radford SJ, Francis ST, Serres S, Greenhaff PL, Moran GW (2021). The aetiology of fatigue in inflammatory bowel disease and potential therapeutic management strategies. Aliment Pharmacol Ther.

[18] Borren N, Tan W, Colizzo FP, Luther J, Garber JJ, Khalili H, van Der Woude CJ, Ananthakrishnan AN (2020). Longitudinal trajectory of fatigue with initiation of biologic therapy in inflammatory bowel diseases: a prospective cohort Study. J Crohns Colitis.

[19] Nocerino A, Nguyen A, Agrawal M, Mone A, Lakhani K, Swaminath A (2020). Fatigue in Inflammatory Bowel Diseases: Etiologies and Management. Adv Ther.

[20] Cairns R, Hotopf M (2005). A systematic review describing the prognosis of chronic fatigue syndrome. Occup Med.

[21] McManimen SL, Sunnquist ML, Jason LA (2019). Deconstructing post-exertional malaise: An exploratory factor analysis. J Health Psychol.

[22] Stussman WA, Snow J, Gavin A, Scott R, Nath A, Walitt B (2020). Characterization of Post-exertional Malaise in Patients With Myalgic Encephalomyelitis/Chronic Fatigue Syndrome. Front Neurol.

[23] Piper BF, Dibble SL, Dodd MJ, Weiss MC, Slaughter RE, Paul SM (1998). The revised piper fatigue scale: psychometric evaluation in women with breast cancer. Oncol Nurs Forum.

[24] Bager P, Vestergaard C, Juul T, Dahlerup JF (2018). Population-based normative data for the inflammatory bowel disease fatigue scale – IBD-F. Scand J Gastroenterol.

[25] Chalder TG, Berelowitz T, Pawlikowska L, Watts S, Wessely D (1993). Development of a fatigue scale. J Psychosom Res.

[26] Donovan KA, Jacobsen PB (2011). The fatigue symptom inventory: a systematic review of its psychometric properties. Support Cancer Ther.

[27] Hann DM, Jacobsen PB, Azzarello LM, Martin SC, Curran SL, Fields KK, Greenberg H, Lyman G (1998). Measurement of fatigue in cancer patients: development and validation of the Fatigue Symptom Inventory. Qual Life Res.

[28] Hann DM, Denniston MM, Baker F (2000). Measurement of fatigue in cancer patients: further validation of the Fatigue Symptom Inventory. Qual Life Res.

[29] Minderhoud IM, Oldenburg B, van Dam PS, van Berge Henegouwen GP (2003). High prevalence of fatigue in quiescent inflammatory bowel disease is not related to adrenocortical insufficiency. Am J Gastroenterol.

[30] Klusmann B, Fleer J, Tovote KA, Weersma RK, van Dullemen HM, Dijkstra G, Schroevers MJ (2021). Trajectories of fatigue in inflammatory bowel disease. Inflamm Bowel Dis.

[31] Becker R, Glauser D (2018). Are prepaid monetary incentives sufficient for reducing panel attrition and optimizing the response rate? An experiment in the context of a multi-wave panel with a sequential mixed-mode design. Bulletin Sociolog Methodology/Bulletin de Méthodologie Sociologique.

[32] Harvey RF, Bradshaw JM (1980). A simple index of Crohn's disease activity. Lancet.

[33] Walmsley RS, Ayres RCS, Pounder RE, Allan RN (1998). A simple clinical colitis activity index. Gut.

[34] French version of FSI instrument [retrieved Oct 13^th^, 2022 from http://www.cas.usf.edu/~jacobsen/Copy%20of%20FSI_final_french.pdf]

[35] Gledhill J, Rodary C, Mahé C, Laizet C (2002). Validation française de l’échelle de fatigue révisée de Piper. Rech Soins Infirm.

[36] Banovic I, Montreuil L, Derrey-Bunel M, Scrima F, Savoye G, Beaugerie L, Gay M-C (2020). Toward Further Understanding of Crohn’s Disease-Related Fatigue: The Role of Depression and Emotional Processing. Front Psychol.

[37] Razadi D, Delvaux N, Farvacques C, Robaye E (1989). Validation of the French version of the HADS in a population of inpatients with cancer. Rev Psychol Appl.

[38] Zigmond AS, Snaith RP (1983). The hospital anxiety and depression scale. Acta Psychiatr Scand.

[39] Loas G, Fremaux D, Marchand MP (1995). Étude de la structure factorielle et de la cohérence interne de la version française de l'échelle d'alexithymie de Toronto à 20 items (TAS-20) chez un groupe de 183 sujets sains. Encéphale.

[40] Bagby RM, Taylor GJ, Parker JD (1994). The twenty-item Toronto alexithymia scale – II convergent, discriminant, and concurrent validity. J Psychosom Res.

[41] MacCallum RC, Browne MW, Sugawara HM (1996). Power analysis and determination of sample size for covariance structure modeling. Psychological Methods.

[42] McIver, J., & Carmines, E. G. (1981). Unidimensional scaling (Vol. 24). sage.

[43] Hu LT, Bentler PM (1999). Cutoff criteria for fit indexes in covariance structure analysis: conventional criteria versus new alternatives. Struct Equ Modelling.

[44] Farrell AM (2010). Insufficient discriminant validity: a comment on Bove, Pervan, Beatty, and Shiu (2009). J Bus Res.

[45] Stover A M,. Reeve BB, Piper BF, Alfano CM, Smith AW, Mitchell SA, Bernstein L, Baumgartner KB, McTiernan A, Ballard-Barbash R. Deriving Clinically Meaningful Cut-scores for Fatigue in a Cohort of Breast Cancer Survivors: a Health, Eating, Activity, and Lifestyle (HEAL) Study. Qual. life Res. 20213;22(9) 10.1007/s11136-013-0360-6. 10.1007/s11136-013-0360-6 PMC3823669 23420495

[46] Banovic I, Gilibert D, Jebrane A, Cosnes J (2012). Personality and fatigue perception in a sample of IBD outpatients in remission: a preliminary study. J Crohns Colitis.

[47] Manning K, Kauffman BY, Rogers AH, Garey L, Zvolensky MJ (2022). Fatigue severity and fatigue sensitivity: relations to anxiety, depression, pain catastrophizing, and pain severity among adults with severe fatigue and chronic low back pain. Behav Med.

[48] Huppertz-Hauss G, Høivik ML, Jelsness-Jørgensen LP, Opheim R, Henriksen M, Høie O, Hovde Ø (2017). Fatigue in a population-based cohort of patients with inflammatory bowel disease 20 years after diagnosis: the IBSEN study. Scand J Gastroenterol.

[49] Wright A, Fisher PL, Baker N, O’Rourke L, Cherry MG (2021). Perfectionism, depression and anxiety in chronic fatigue syndrome: a systematic review. J Psychosom Res.

[50] McTaggart-Cowan H, King MT, Norman R, Costa DSJ, Pickard AS, Viney R, Stuart J, Peacock SJ (2022). The FACT-8D, a new cancer-specific utility algorithm based on the Functional Assessment of Cancer Therapies-General (FACT-G): a Canadian valuation study. Health Qual Life Outcomes.

[51] Vlaeyen JW (2015). Learning to predict and control harmful events: chronic pain and conditioning. Pain.

[52] Ali S, Adamczyk L, Burgess M, Chalder T (2019). Psychological and demographic factors associated with fatigue and social adjustment in young people with severe chronic fatigue syndrome/Myalgic encephalomyelitis: a preliminary mixed-methods study. J Behav Med.

[53] Augustinova M, Clarys D, Spatola N, Ferrand L (2018). Some further clarifications on age-related differences in Stroop interference. Psychon Bull Rev.

[54] Burca M, Chausse P, Ferrand L, Parris BA, Augustinova M (2022). Some further clarifications on age-related differences in the Stroop task: new evidence from the two-to-one Stroop paradigm. Psychon Bull Rev.

[55] Parris BA, Hasshim N, Wadsley M, Augustinova M, Ferrand L (2022). The loci of Stroop effects: a critical review of methods and evidence for levels of processing contributing to color-word Stroop effects and the implications for the loci of attentional selection. Psychol Res.

